# Wnt/β-catenin pathway transactivates microRNA-150 that promotes EMT of colorectal cancer cells by suppressing CREB signaling

**DOI:** 10.18632/oncotarget.9893

**Published:** 2016-06-07

**Authors:** Yan-Hua Guo, Lu-Qin Wang, Bin Li, Hui Xu, Jian-Hua Yang, Li-Si Zheng, Peng Yu, Ai-Dong Zhou, Yin Zhang, Shu-Juan Xie, Zi-Rui Liang, Chen-Min Zhang, Hui Zhou, Qu Liang-Hu

**Affiliations:** ^1^ Key Laboratory of Gene Engineering of the Ministry of Education, State Key Laboratory of Biocontrol, School of Life Sciences, Sun Yat-Sen University, Guangzhou, P. R. China; ^2^ Present address: Department of Neurosurgery, The University of Texas MD Anderson Cancer Center, Houston, Texas, USA; ^3^ Present address: Guangzhou Quality Supervision and Testing Institute, Guangzhou, China

**Keywords:** Wnt/β-catenin, miR-150, CREB, EMT, colorectal cancer

## Abstract

A hallmark of aberrant activation of the Wnt/β-catenin signaling pathway has been observed in most colorectal cancers (CRC), but little is known about the role of non-coding RNAs regulated by this pathway. Here, we found that miR-150 was the most significantly upregulated microRNA responsive to elevated of Wnt/β-catenin signaling activity in both HCT116 and HEK293T cells. Mechanistically, the β-catenin/LEF1 complex binds to the conserved TCF/LEF1-binding element in the miR-150 promoter and thereby transactivates its expression. Enforced expression of miR-150 in HCT116 cell line transformed cells into a spindle shape with higher migration and invasion activity. miR-150 markedly suppressed the CREB signaling pathway by targeting its core transcription factors CREB1 and EP300. Knockdown of CREB1 or EP300 and knockout of CREB1 by CRISPR/Cas9 phenocopied the epithelial-mesenchymal transition (EMT) observed in HCT116 cells in response to miR-150 overexpression. In summary, our data indicate that miR-150 is a novel Wnt effector that may significantly enhance EMT of CRC cells by targeting the CREB signaling pathway.

## INTRODUCTION

The Wnt/β-catenin signaling pathway plays a central role in normal embryogenesis and development as well as in various human diseases. [1–3] Dysregulation of Wnt/β-catenin signaling is involved in multiple tumors and regulates various processes, including tumor initiation, tumor growth and metastasis. [4] Particularly, it is reported that approximately 90% of sporadic colorectal cancers (CRC) have an activation mutation Wnt/β-catenin pathway. [5] Upon activation of Wnt signaling, β-catenin accumulates in the cytoplasm and then translocates into the nucleus, where it associates with members of the TCF/LEF1 transcription factor family to modulate the transcription of target genes that mediate the widespread effects of Wnt signaling on cellular behavior.

Since the discovery of the proto-oncogene MYC as a direct transcriptional target of Wnt/β-catenin signaling in 1998, more than one hundred protein-coding genes have been determined to be direct targets of the Wnt pathway. [6] In addition to a handful of genes that are directly suppressed by the β-catenin/TCF/LEF1 transcription factor complex, the majority of Wnt target genes are trans-activated by the Wnt signaling pathway and thus serve as pathway effectors. Among these Wnt effectors, which include c-Myc, ZEB1 and Twist, [7–9] are transcription factors that can in turn modulate the transcription of numerous downstream genes and transform the Wnt signals into a whole genome response. MicroRNAs (miRNAs) are endogenous small non-coding RNAs that suppress protein expression by destabilizing messenger RNAs (mRNAs) or by inhibiting their translation. [10] Studies over the past decade have uncovered a recurring paradigm, namely, that miRNAs are key regulators of cell fate and behavior under various physiological and pathological conditions. [11, 12] On one hand, just like transcription factors, a single miRNA has the ability to directly control the expression of hundreds of genes. On the other hand, unlike transcription factors that regulate gene expression at transcriptional level, miRNAs directly inhibit the protein synthesis of existing mRNAs and thus monitor and fine-tune the translation of hundreds of proteins in real-time. These characteristics of miRNAs make them ideal effectors of signaling pathways that regulate global cellular behavior in a previously unknown mechanism. Recently, a handful of miRNAs have been shown to be directly transactivated by the β-catenin/TCF/LEF1 transcription factor complex, [13–18] showing miRNAs as a new class of Wnt effector that contribute significantly to the regulatory role of Wnt/β-catenin pathway. However, with comparison to the numerous protein effectors, the identity of Wnt miRNAs and their functions in cancers remain largely undetermined.

In this study, we investigated the transcriptional regulation and function of miR-150-5p (referred to as miR-150) that was the most significantly responsive to the activation of Wnt/β-catenin signaling pathway in the LiCl-treated CRC cells. We showed that miR-150 was directly transactivated by β-catenin/LEF1, and overexpression of miR-150 significantly promoted CRC cell migration *in vitro* and *in vivo*. These results set miR-150 as an effector of Wnt/β-catenin signaling in EMT and the malignant progression of CRC.

## RESULTS

### A group of miRNAs were responsive to the activation of Wnt/β-catenin signaling

**Figure 1:**
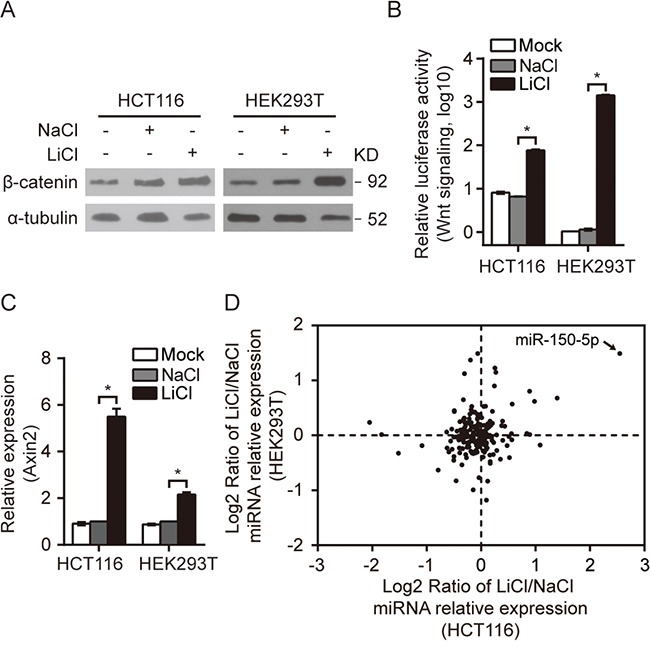
A group of miRNAs were responsive to the activation of Wnt/β-catenin signaling **A-C.** β-catenin Western blot (A), Wnt pathway reporter assay (B) and Axin2 qRT-PCR (C) in HCT116/HEK293T cells treated with LiCl as indicated. The same molarity of NaCl was used as a negative control (NC) for LiCl stimulation. **D.** The relative expression of miRNAs conserved between humans and mice were analyzed by qRT-PCR in LiCl-treated HCT116 and HEK293T cells; miR-150-5p is indicated by an arrow. All qRT-PCR experiments were performed at least three times. Error bars represent SEM. *p< 0.05 by Student's *t*-test.

To identify miRNAs that are responsive to the Wnt signaling pathway, we initiated the study by treating HCT116 and HEK293T cells with LiCl to activate the Wnt/β-catenin signaling pathway. LiCl is commonly used to mimic the activity of Wnt/β-catenin signaling via inhibiting GSK-3β mediated β-catenin phosphorylation. [19, 20] HCT116 cells have both mutant and wild-type β-catenin alleles, but the mutant allele does not confer higher Wnt activity. [21] In contrast, HEK293T cells possess an intact Wnt/β-catenin pathway but exhibit low Wnt activity. As expected, the Wnt/β-catenin pathway was activated in response to LiCl treatment in both HCT116 and HEK293T cells, as indicated by the significant increases in β-catenin protein levels, in Wnt reporter activities and in the expression of the Wnt target gene Axin2 (Figure 1A, 1B and 1C).

A miRNA qRT-PCR array capable of monitoring the expression of 212 highly conserved (between human and mouse) miRNAs was utilized to investigate the miRNA expression profile in LiCl-treated HCT116 and HEK293T cells (Supplementary Table S6). This survey identified 16 miRNAs in HCT116 cells and 17 miRNAs in HEK293T cells with significant changes in expression (either 1.5-fold upregulated or 1.5-fold downregulated) (Figure 1D, Table 1). Among these miRNAs, miR-150-5p, miR-34b-5p, miR-34c-5p and miR-302b-3p were up-regulated and miR-379-5p was down-regulated in both Wnt-activated HCT116 and HEK293T cells. Remarkably, miR-150-5p ranked at the top of the upregulated miRNAs in both cell lines (Figure 1D, Table 1). In addition, we treated cells with another Wnt modulator BIO. As expected, the Wnt/β-catenin pathway was activated in response to BIO treatment in both HCT116 and HEK293T cells, (Supplementary Figure S1A, S1B). We discovered that activation of Wnt signaling by BIO also cause significant upregulation of miR-150-5p by qRT-PCR assay (Supplementary Figure S1C).

**Table 1:** Differential miRNA expression in HCT116 and HEK293T cells in response to LiCl

miRNA	HCT116-FoldChange	miRNA	HEK293T-FoldChange
miR-150-5p	5.845	miR-150-5p	2.809
miR-34b-5p	2.634	miR-146b-5p	2.807
miR-302a-3p	2.122	miR-146a-5p	2.588
miR-34c-5p	1.974	miR-142-3p	2.337
miR-302d-3p	1.861	miR-139-5p	2.214
miR-302b-3p	1.854	miR-199a-5p	2.179
miR-367-3p	1.793	miR-132-3p	1.865
miR-495-3p	0.663	miR-302b-3p	1.745
miR-33a-5p	0.648	miR-34b-5p	1.602
miR-221-3p	0.648	miR-199a-3p	1.568
miR-208b-3p	0.645	miR-34c-5p	1.536
miR-379-5p	0.580	miR-379-5p	0.645
miR-382-5p	0.472	miR-381-3p	0.586
miR-299-5p	0.349	miR-127-3p	0.564
miR-411-3p	0.281	miR-148b-3p	0.561
miR-410-3p	0.241	miR-137	0.503
		miR-369-5p	0.441

### miR-150 is directly transactivated by β-catenin/LEF1

**Figure 2:**
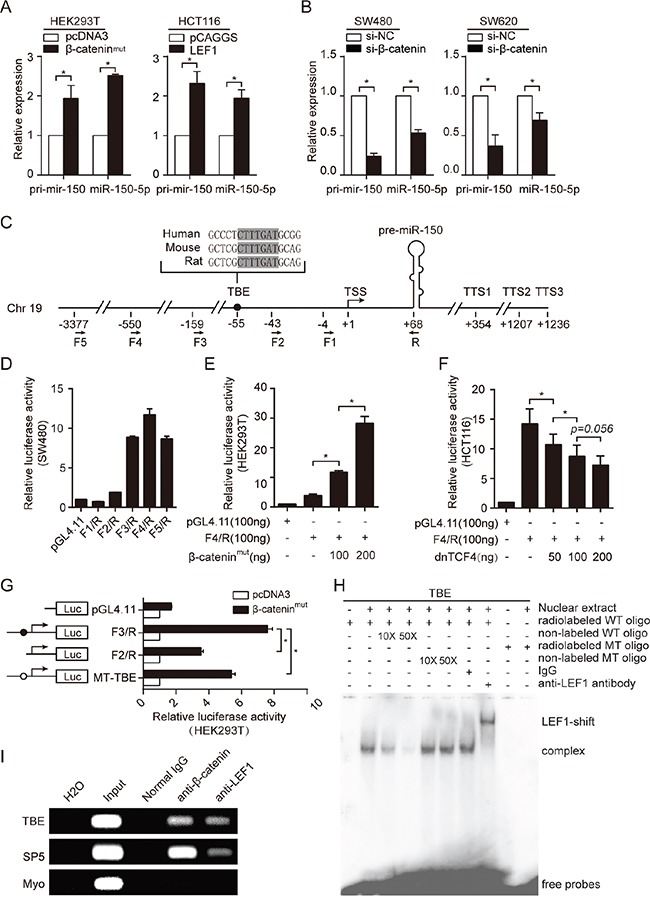
miR-150 is directly transactivated by β-catenin/LEF1 **A.** qRT-PCR for pri-mir-150 and miR-150 expression after overexpression of β-catenin (HEK293T cells) or LEF1 (HCT116 cells). **B.** qRT-PCR for pri-mir-150 and miR-150 expression after siRNA-mediated β-catenin knockdown in SW480 and SW620 cells. **C.** Schematic of the genomic miR-150 region. The transcriptional start site (TSS) is marked as +1, and the three transcription termination sites (TTSs) are indicated. The sequence and location of the TCF/LEF1 binding site (TBE) is be noted by a black dot. The locations of the primers used in this study to generate plasmid constructs are indicated by arrows below the schematic. **D.** Deletion analysis of the miR-150 gene promoter in SW480 cells. The luciferase activity of each reporter construct is presented relative to that of the pGL4.11-basic vector. **E, F.** Luciferase reporter assays showing the dose-dependent effect of β-catenin (left panel) or dnTCF4 (right panel) overexpression on miR-150 promoter activity. **G.** Luciferase reporter assays showing the effect of β-catenin on the activity of the deleted or mutated TBE promoter. **H.** Gel shift and supershift assays showing binding of LEF1 to the miR-150 promoter in SW480 cells. 10 or 50-fold molar excess of unlabeled wild-type (WT) cold probe or mutate-type (MT) cold probe was used in the competition EMSA assay. **I.** Chromatin immunoprecipitation (ChIP) assays demonstrating an *in vivo* interaction between the β-catenin/LEF1 complex and the miR-150 promoter. The TBE site in the SP5 gene promoter was used as a positive control, and the coding region of Myo was used as a negative control (NC). All experiments were repeated at least three times with similar results. Error bars represent SEM. *p < 0.05 by Student's *t*-test.

To verify whether the LiCl and BIO-induced miR-150 upregulation was a specific result of the activation of Wnt/β-catenin signaling, we assessed the effects of BIO, β-catenin and LEF1 on the expression of the primary miR-150 transcript (pri-mir-150) and mature miR-150. Overexpression of mutant-activated β-catenin (β-catenin^mut^) in HEK293T cells or of LEF1 in HCT116 cells significantly increased miR-150 expression (Figure 2A, Supplementary Figure S1C, S1D). Conversely, siRNA-mediated knockdown of β-catenin in SW480 and SW620 cells reduced the expression levels of both pri-mir-150 and miR-150 (Figure 2B, Supplementary Figure S1E).

To further explore the relationship between miR-150 and the Wnt/β-catenin pathway, we analyzed the endogenous levels of miR-150 and Wnt/β-catenin signaling activity in different CRC cell lines and in HEK293T cells. As shown in Supplementary Figure S1F, S1G and S1H, miR-150 expression positively correlated with Wnt/β-catenin pathway activity in most of these cell lines, suggesting that the Wnt/β-catenin signaling pathway may regulate miR-150 in a wide variety of cells.

To investigate the mechanism by which Wnt/β-catenin signaling is involved in the transcriptional regulation of miR-150, we analyzed the promoter region of miR-150. Because the transcriptional start site (TSS) and transcriptional termination site (TTS) for miR-150 were unknown, we first identified the 5′ and 3′ ends of the miR-150 primary transcript by Rapid Amplification of cDNA Ends (RACE). As shown in Figure 2C and Supplementary Figure S2A and S2B, a single TSS and three TTSs were identified.

To determine whether the 5′ flanking region of miR-150 contained a functional promoter, we performed a unidirectional deletion analysis on the approximately 3.4 kb DNA fragment. All the deletion derivatives were cloned into the pGL4.11-basic vector, and luciferase activity was measured in SW480 cells, which harbor a highly activated Wnt/β-catenin signaling pathway. As shown in Figure 2D, fragment F4/R exhibited the highest activity, suggesting the presence of the core promoter in this region. To further determine whether the F4/R fragment contained a Wnt-responsive element, the F4/R construct was co-transfected into HEK293T cells with the β-catenin plasmid or into HCT116 cells with a dominant-negative TCF4 (dnTCF4) plasmid. As expected, the F4/R fragment responded to β-catenin-mediated activation or dnTCF4-mediated inhibition in a dose-dependent manner (Figure 2E and 2F).

Studies have proven that the β-catenin/TCF/LEF1 complex regulates the transcriptional activity of target genes by binding to the consensus core TCF/LEF1-binding site (5′-A/TA/TCAAAG-3′) within promoter regions. [22, 23] To precisely identify the β-catenin/TCF/LEF1 binding sites that orchestrated miR-150 expression, we scanned the 5′ flanking region of the miR-150 gene using a computational method called ACTLocater, which identifies accessible and conserved transcription factor binding sites based on data released by the ENCODE Project. [24] Interestingly, one perfect TCF/LEF1-binding site (TBE, 5′-CTTTGAT-3′) was found 55 nucleotides upstream of the TSS of miR-150 (Figure 2C). To confirm whether the predicted site was directly bound by the β-catenin/TCF/LEF1 complex and whether it contributed to Wnt-induced miR-150 upregulation, we generated a construct with a mutant TBE (MT-TBE) and then compared its activity with the F3/R construct, which contains a shorter 5′ flanking fragment but harbors the entire TBE, and with the F2/R construct, which lacks the TBE. As shown in Figure 2G, deletion or mutation of the TBE in the miR-150 promoter significantly reduced the responsiveness to β-catenin overexpression, suggesting that this site was required for the trans-activation of miR-150 by Wnt/β-catenin signaling.

To establish binding of LEF1 or TCF to the TBE *in vitro*, electrophoretic mobility-shift assays (EMSA) were performed by using SW480 cells, which express high endogenous LEF1 and β-catenin (Supplementary Figure S1H). A shift band was detected when incubated the labeled wild-type (WT) oligonucleotide containing TBE with nuclear extract from SW480 cells. Competition EMSA and antibody supershift assays confirmed that complexes are specific and the shift band was mainly composed of LEF1 (Figure 2H). We further performed chromatin immunoprecipitation (ChIP) experiments. As demonstrated in Figure 2I, both β-catenin and LEF1 occupied the TBE site in the endogenous miR-150 promoter as well as the SP5 promoter (positive control) but not the Myo ex2 promoter (negative control). [25, 26] Collectively, these results indicated that miR-150 is a bona fide target of the Wnt/β-catenin signaling pathway.

### Ectopic expression of miR-150 promoted EMT, migration and invasion of HCT116 cells

**Figure 3:**
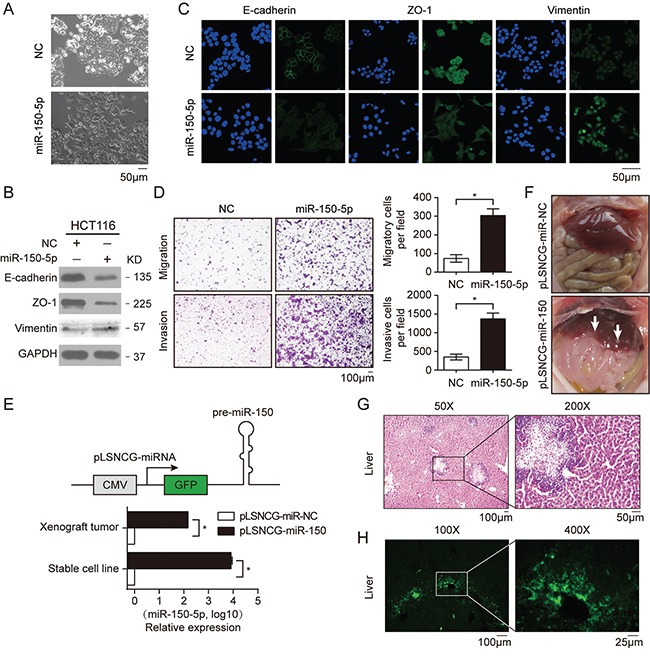
Ectopic expression of miR-150 promoted EMT, migration and invasion of HCT116 cells **A.** Morphological changes in HCT116 cells transfected with miR-150-5p mimics or NC after 48 h. **B.** Western blotting for EMT markers (adherens junction protein E-cadherin, tight-junction protein ZO-1 and mesenchymal intermediary filament vimentin) in HCT116 cells treated with miR-150-5p mimics. **C.** Immunofluorescent microscopy analysis of the localization and expression of EMT markers in HCT116 cells. Cells were counterstained with Hoechst 33342 dye. Scale bars, 50 μm. **D.** Migration and invasion assays in HCT116 cells treated with miR-150-5p mimics. Representative images are shown in the left panel. The mean number of cells per visual field was determined while viewing the entire chamber, and the experiments were performed in triplicate (right panel). Scale bars, 100 μm. **E.** A schematic of the structure of the plasmid for lentiviral overexpression of miR-150 (upper panel). qRT-PCR analysis of miR-150 expression in HCT116 stable cell lines and xenograft tumors (lower panel). Experiments were performed in triplication. Error bars represent SEM. *p< 0.05 by Student's *t*-test. **F.** Photographs of mice injected with HCT116-pLSNCG-miR-150 cells illustrate the metastatic sites found. The white arrow indicates clusters of metastatic cells. **G, H.** Microscopic images of HE staining (G) and GFP expression (H) in livers isolated from mice 35 days after the subcutaneous injection of HCT116 cells stably overexpressing miR-150. Scale bars, 100 μm.

To investigate the biological role of miR-150 in CRC tumorigenesis and progression, we first performed a transient overexpression study in HCT116 cells using miR-150-5p mimics. Interestingly, we found that miR-150 induced an obvious morphological change in HCT116 cells, from a round epithelial-like shape to a spindle shape; this phenotypic change implied that the cells may have undergone the epithelial-mesenchymal transition (EMT) (Figure 3A). To validate this hypothesis, we detected the protein levels of EMT markers in these cells. Both western blotting and immunofluorescence data showed that miR-150 overexpression markedly repressed E-cadherin and ZO-1 expression and enhanced vimentin protein expression, further indicating that miR-150 induced EMT in HCT116 cells (Figure 3B and 3C). Conversely, we found that expression of E-cadherin and ZO-1 was up-regulated by miR-150 inhibitor in SW480 cells (Supplementary Figure S3A). Given that EMT is usually associated with increased migratory ability, we performed transwell assays. As shown in Figure 3D and Supplementary Figure S3B, transfection of miR-150 into HCT116 cells significantly promoted cellular invasion and migration, while miR-150 inhibitors blocked TGF-β mediated migration and invasion of SW480 cells.

To further substantiate the role of miR-150 *in vivo*, we established a HCT116 cell line stably overexpressing miR-150 (HCT116-pLSNCG-miR-150, Figure 3E) using a lentiviral system as described previously [27] and the cells were then subcutaneously implanted into BALB/c nude mice. Quantitative RT-PCR data revealed that miR-150 expression was significantly increased in both cultured cells and xenograft tumors relative to the control (pLSNCG-miR-NC) (Figure 3E). Ten mice were used in each group and sacrificed 35 days after inoculation of tumor cells. Three of ten mice of miR-150 overexpression group showed signs of hepatic metastasis, but no sign was found in NC-group (Figure 3F). Moreover, HE staining and GFP immunofluorescence of the liver sections confirmed that the cells populating the metastatic nodules had migrated from the xenografted HCT116 cells (Figure 3G and 3H). However, miR-150 had no impact on xenograft growth, which was in line with the effect of overexpression of miR-150 on HCT116 cells proliferation *in vitro* (Supplementary Figure S3C and S3D). These results indicated that miR-150 significantly increases CRC metastasis *in vivo*.

As activation of Wnt pathway is reported to promote EMT in cancer cells, [5, 8, 9, 28] we confirmed this by activating Wnt pathway using LiCl treatment or LEF1 overexpression in HCT116 cells and detected decreased expression of epithelial markers E-cadherin and ZO-1 (Supplementary Figure S3E and S3F). Furthermore, we activated Wnt signaling in HCT116 cells using LiCl and at the same time inhibit the expression miR-150 by tranfecting miR-150-inhibitor. We found that inhibition of miR-150 attenuate the effect of enhanced migration and invasion caused by activation of Wnt signaling (Supplementary Figure S3G). Therefore, these results indicated that Wnt-transactivated miR-150 contributed to the effects of aberrant activation of the Wnt/β-catenin signaling pathway in CRC cells.

### miR-150 suppressed CREB signaling by directly targeting EP300 and CREB1

**Figure 4:**
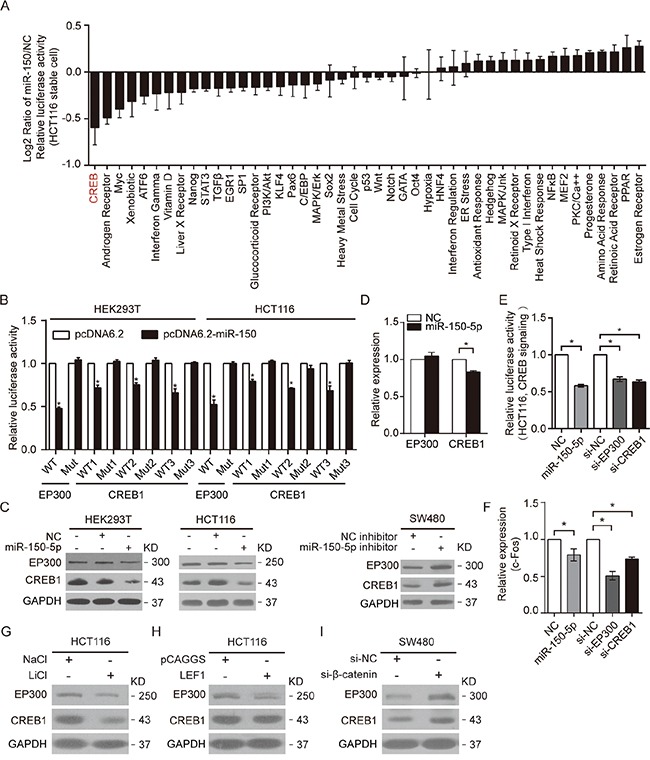
miR-150 suppressed CREB signaling by directly targeting EP300 and CREB1 **A.** The 45-pathway assay revealed the effects of miR-150 on the activity of various pathways in HCT116 stable cell lines. The data were log2-transformed. **B.** Relative luciferase activity of WT and Mut EP300 and CREB1 reporters in HEK293T and HCT116 cells after co-transfection with the miR-150 expression vector or an empty vector. **C.** Western blotting for EP300 and CREB1 expression in HEK293T and HCT116 cells after transfection with miR-150-5p mimics or NC, or in SW480 cells after transfection with miR-150-5p inhibitors or NC. **D.** The mRNA levels of EP300 and CREB1 were determined by qRT-PCR after transfecting HCT116 cells with miR-150-5p mimics. **E, F.** The activity of CREB signaling and mRNA levels of c-Fos were measured by luciferase assay and qRT-PCR in HCT116 cells transfected with the miR-150-5p mimics, EP300 siRNA or CREB1 siRNA. **G, H.** Western blot analysis showing the effects of activation of Wnt signaling by LiCl treatment or LEF1 overexpression on EP300 and CREB1 expression in HCT116 cells. **I.** Western blot analysis showing the effects of knockdown of Wnt signaling by special siRNA on EP300 and CREB1 expression in SW480 cells. For D and F, the RT-PCR experiments were performed three times with similar results. Error bars represent SEM. *p< 0.05 by Student's *t*-test.

To explore the molecular mechanism by which miR-150 promoted CRC metastasis, we employed two strategies to identify the functional targets of miR-150 (Supplementary Figure S4A). We searched for the predominant signaling pathways that were regulated by miR-150 in HCT116 stable cells using a Cignal 45-Pathway Reporter Array. As shown in Figure 4A, CREB signaling was the most significantly downregulated pathway in HCT116-pLSNCG-miR-150 cells compared with negative control cells, suggesting that miR-150 may target the critical mediators of this pathway. After analyzing the genes predicted to be involved in the CREB pathway, we obtained 161 candidate genes. We also used computational tools (TargetScan and miRanda [30, 31]) to predict miR-150 targets; with these methods, we obtained 4327 candidate genes. By comparing the two pools of predicted target genes, we identified 34 candidate genes that were included in both pools (Supplementary Figure S4A). Interestingly, CREB1, the central transcription factor of the CREB pathway, was a predicted miR-150 target. In addition, EP300, which can act as a co-activator in the CREB pathway, has been reported to be regulated by miR-150 in high glucose-induced cardiomyocyte hypertrophy. [32]

To determine whether CREB1 and EP300 were direct targets of miR-150, we synthesized 3′UTR fragments of CREB1 and EP300 harboring either wild-type (WT) or mutant (Mut) putative binding motifs for miR-150 and inserted them downstream of the Renilla luciferase gene in the psiCHECK-2 vector (Supplementary Figure S4B). The 3′UTR reporter assays revealed that miR-150 overexpression significantly attenuated the activity of Renilla luciferase downstream of the wild-type 3′UTRs of CREB1 and EP300, whereas the mutant 3′UTRs abrogated the miR-150-induced repression (Figure 4B). Correspondingly, clear reductions in endogenous CREB1 and EP300 protein expression were observed in HEK293T and HCT116 cells transfected with miR-150 mimics. Conversely, expression of CREB1 and EP300 was up-regulated by transient transfection of SW480 cells using miR-150 inhibitors (Figure 4C). Notably, CREB1 mRNA levels were also significantly decreased by miR-150 transfection in HCT116 cells (Figure 4D).

To determine whether the repression of CREB1 and EP300 accounted for the miR-150-mediated downregulation of the CREB pathway, we analyzed the effects of EP300 and CREB1 knockdown on the CREB pathway. As expected, HCT116 cells transfected with siRNA against EP300 or CREB1 exhibited decreased CREB pathway activity, similar to the effect of miR-150 overexpression (Figure 4E, Supplementary Figure S4C). Moreover, c-Fos, which is a downstream target gene of CREB signaling, [33] was significantly downregulated when HCT116 cells were transiently transfected with miR-150-5p mimics, EP300 siRNA or CREB1 siRNA (Figure 4F). Together, these results indicated that miR-150 regulated the CREB pathway by directly targeting CREB1 and EP300.

Importantly, we observed that activating Wnt pathway by LiCl treatment or LEF1 overexpression in HCT116 cells caused the decrease of CREB1 and EP300 expression, while knockdown β-catenin in SW480 cells had opposite effects, indicating that Wnt pathway could suppress the expression of these two targets (Figure 4G, 4H and 4I). Therefore, these results indicated that Wnt-transactivated miR-150 suppressed CREB pathway by directly targeting CREB1 and EP300 in CRC cells.

### CREB1 and EP300 were the key mediators of miR-150-regulated EMT and CRC cell migration

**Figure 5:**
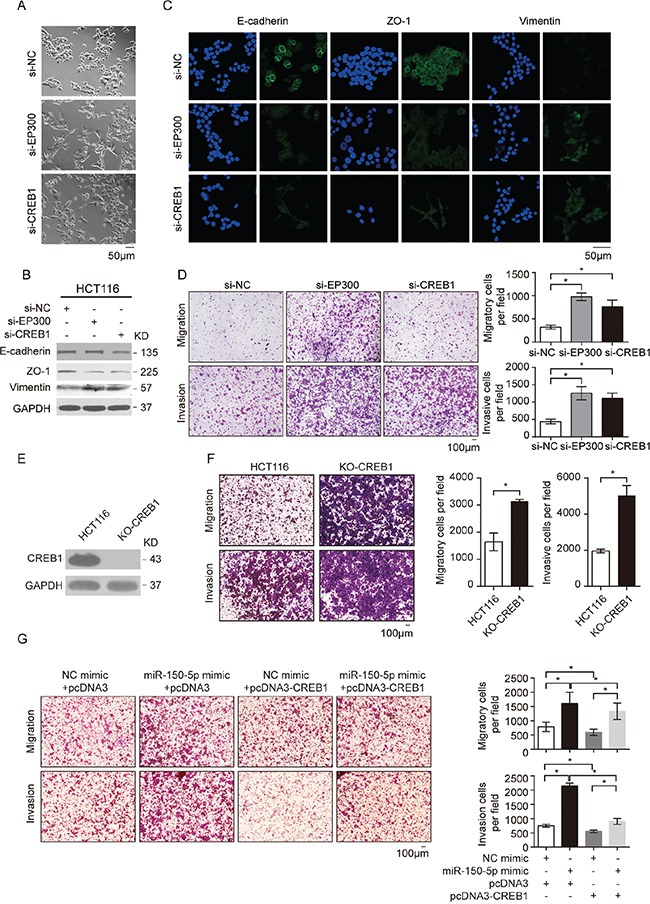
CREB1 and EP300 were the key mediators of miR-150-regulated EMT and CRC cell migration **A.** Morphological changes in HCT116 cells 48 h after transfection with siRNA against EP300 or CREB1 or with NC. Scale bars, 50 μm. **B.** Western blotting for EMT markers (E-cadherin, ZO-1 and vimentin) in HCT116 cells treated with siRNA against EP300 or CREB1 or with NC. **C.** Immunofluorescent microscopy analysis of the localization and expression of EMT markers in HCT116 cells. Cell nuclei were labeled with Hoechst 33342 dye. Scale bars, 50 μm. **D.** Migration and invasion assays were performed in HCT116 cells after transfection with NC, si-EP300 and si-CREB. **E.** Western blotting for CREB1 expression in HCT116 cells after knockout by CRISPR/Cas9. **F.** Migration and invasion assays were performed in HCT116 cells after knockout CREB1 by CRISPR/Cas9. **G.** Transwell migration and invasion assays using HCT116 cells after co-transfection with miR-150-5p mimics and pcDNA3-CREB1, and NC. For D, F and G, representative images are shown in the left panel. The mean number of cells per visual field was determined in three randomly selected visual fields per chamber (right panel). The data were representative of three independent experiments. Error bars represent SEM. *p < 0.05 by Student's *t*-test.

The above results prompted us to determine whether the downregulation of CREB signaling mediated the effects of miR-150 overexpression: EMT and the subsequent increased migration of CRC cells. As expected, knockdown of EP300 or CREB1 by siRNA resulted in a similar mesenchymal-like morphological change in HCT116 cells (Figure 5A). Consistent with this phenotype, the analysis of E-cadherin, ZO-1 and vimentin expression revealed that EP300 and CREB1 knockdown mimicked the effects of miR-150 overexpression in HCT116 cells (Figure 5B and 5C). In addition, the invasion and migration of HCT116 cells were significantly elevated by EP300 or CREB1 knockdown but were reduced by CREB1 overexpression (Figure 5D, Supplementary Figure S5A and S5B). To further exclude the possibility that the phenotype is caused by off-target effects of siRNAs, we completely knockout CREB1 in HCT116 cells by CRISPR/Cas9 and detected a consistent phenotype of enhanced migration and invasion (Figure 5E and 5F). Importantly, CREB1 overexpression attenuated the migration- and invasion-promoting effects of miR-150 (Figure 5G). Therefore, these data strongly indicated that both CREB1 and EP300 were critical targets of miR-150, and by targeting them, miR-150 suppressed CREB signaling and induced EMT in CRC cells. It is worth noting that c-Myb, the known target of miR-150 in lymphocytes, was not repressed by the overexpression of miR-150 in the HCT116 cells (data not shown). Both target dual luciferase assay and western blot confirmed this result, suggesting that miR-150 functions in a context-dependent manner.

## DISCUSSION

**Figure 6:**
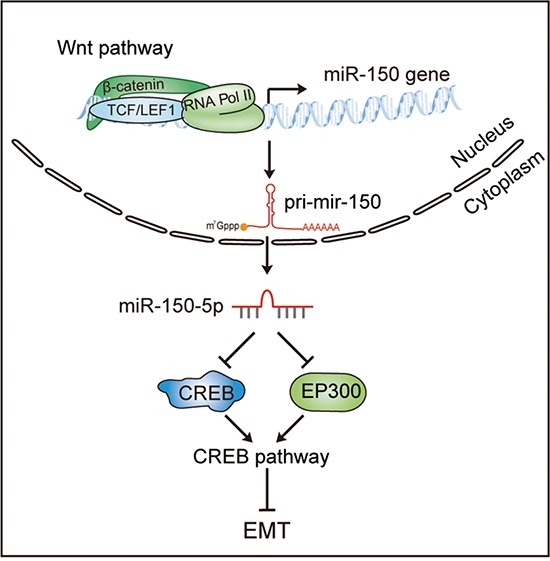
A model of the Wnt/β-catenin-miR-150-CREB signaling regulation axis in colorectal cancer The Wnt/β-catenin signaling pathway transcriptionally activates the expression of miR-150, and miR-150-5p subsequently suppresses the CREB pathway by directly targeting EP300 and CREB1, thereby inducing EMT in CRC cells.

In the present study, we demonstrated an interesting miRNA effector of Wnt signaling, miR-150, that plays a central role in mediating the crosstalk between the Wnt/β-catenin and CREB signaling pathways and contributes to the EMT of CRC cells (Figure 6). According to our model, in CRC cells with activated Wnt signaling, β-catenin/LEF1 transactivates miR-150 by directly binding to its promoter, and the increased miR-150 expression in turn suppresses CREB signaling by targeting CREB1 and EP300. Ultimately, the downregulation of the CREB signaling pathway results in EMT and thus facilitates CRC cell invasion and migration. This model can explain the abnormal expression of miR-150 in various cancers with activated Wnt pathway.

miR-150 was originally found to be specifically and highly expressed in mature B and T cells, where it plays critical roles in normal hematopoiesis and immunity. [34, 35] Although miR-150 is expressed at much lower levels in other tissues under normal physical conditions, [34] later studies suggested that miR-150 is dysregulated in human solid tumors and involves in the development or/and progression of many types of cancer. [29, 36–45] In this study, we provided direct evidence that miR-150 plays a role in regulating CRC cell EMT, invasion and migration. We have also found that miR-150 increased the migration of RKO cells (Supplementary Figure S6A and S6B). Collectively, our data clearly indicated that miR-150 may have the effect of pro-migration and contribute to the development of CRC. Furthermore, we demonstrated that activation of the Wnt/β-catenin signaling in HCT116 cells resulted in reduction of E-cadherin and ZO-1, which is in agreement with previous studies that the Wnt/β-catenin pathway contributed to EMT, migration and invasion of cells, [5, 8, 9, 28] suggesting that Wnt/β-catenin signaling may contribute to the development of cancers depending on the coordinated regulation between its downstream non-coding RNA and protein coding genes.

From the 45-pathway reporter array analysis, we found that miR-150 overexpression seriously affects multiple signaling pathways for cell growth or proliferation, and CREB was the most downregulated. Importantly, we found that activation of Wnt/β-catenin pathway in HCT116 cells suppressed CREB signaling pathway core factors EP300 and CREB. These findings revealed an unexpected significance of the CREB pathway in colorectal cancer biology, providing evidence in understanding CREB signaling from a new perspective. The CREB signaling pathway participates in various biological processes, [46] including cell growth, differentiation, and metabolism [47] as well as neuronal activity [48] and immune function. [49] In some cases, CREB is considered to be an oncogenic transcription factor because it is overexpressed and/or constitutively phosphorylated in several human cancers and induces a cell growth and antiapoptotic survival signal. [50] However, other reports have shown that CREB suppresses tumorigenesis, particularly, in inhibiting the invasion and migration of pancreatic and breast cancer cells. [51, 52] Intriguingly, EP300, a transcriptional co-activator of CREB1, is frequently mutated, lost or underexpressed in numerous types of cancer, such as gastric cancer, colon cancer, and breast cancer. [53, 54] Krubasik *et al*. reported that disrupting EP300 in HCT116 cells resulted in EMT and migration. [55] These findings indicate that EP300, a known target of miR-150, [32] may act as a tumor suppressor in cancers. In the present study, we showed that knockdown of EP300 or CREB1 promoted EMT in HCT116 cells and increased the invasion and migration of these cells, whereas CREB1 overexpression had the opposite effects. Furthermore, we completely knockout CREB1 in HCT116 cells using CRISPR/Cas9 and observed the similar effects, strongly suggesting that CREB pathway plays a role in the development of CRC. Although it is currently unclear how CREB signaling regulates EMT in CRC cells and subsequent migration, several previous studies have suggested that CREB signaling influences TGF-β signaling in pancreatic cancer cells and fibroblast [51, 56] and that EP300 can act as a transcriptional co-activator to regulate E-cadherin expression. [57] It will be interesting to further explore the underlying mechanism by which CREB signaling regulates EMT in CRC cells and affects their migration.

Previously, we reported that Wnt/β-catenin signaling can directly transactivate the miR-372/373 cluster in CRC cells to promote cell proliferation and invasion, in part by targeting the Wnt signaling inhibitor Dickkopf-1 (Dkk1). [14] Recently, miR-146a was determined to be transactivated by the β-catenin/TCF4 complex in CRC cells, in which miR-146a directed the symmetric division of colorectal cancer stem cells by targeting NUMB, a protein that controls β-catenin stability by promoting its polyubiquitylation. [17] Both the miR-372/373 cluster and miR-146a maintain or enhance Wnt signaling via a similar positive feedback mechanism. However, miR-150 mediates the crosstalk between Wnt and other signaling pathways. The Wnt-transactivated miRNAs (designated as WntmiR) may play essential roles in the oncogenesis of CRC mediated by the aberrant activation of Wnt/β-catenin signaling through various mechanisms.

In addition to miR-150, a cohort of miRNAs have been upregulated following the activation of Wnt/β-catenin signaling in our cell models (see Table 1), and there are WntmiRs remained to be identified in different cells. [58, 59] Thus, WntmiR may consist of numerous miRNAs and play essential roles in maintaining the robustness and transduction of Wnt signaling. Furthermore, as the same pathway may activate different effectors in a context-dependent manner, we can speculate that the combinatorial use of a subset of WntmiRs accounts for the unique regulation of gene expression by Wnt signaling in response to different stimuli and in the cancer therapy.

## MATERIALS AND METHODS

### Cell cultures and treatments

Cell lines derived from the human CRC (SW480, SW620, HCT116 and RKO) and HEK293T were all purchased from the Shanghai Institute of Cell biology, Chinese Academy of Science. HEK293FT cells were obtained from Invitrogen (Carlsbad, CA, USA). Cell lines were maintained using standard media and conditions. Establishment of HCT116 subline that stably expressed miR-150 was performed as previously described. [27] To stimulate the Wnt pathway by LiCl, HEK293T cells were treated with 30 mM LiCl (Sigma-Aldrich, L9650) for 12 h and HCT116 cells were treated with 20 mM LiCl for 24 h, and negative control cells were treated with NaCl (Sigma-Aldrich, S5886) at the same time. To stimulate the Wnt pathway by BIO, HEK293T cells and HCT116 cells were treated with 2uM BIO (Sigma-Aldrich, B1686) for 24 h, and negative control cells were treated with DMSO (Sigma-Aldrich) at the same time. In order to activate EMT programs, SW480 cells were treated with 20 ng/ml TGFβ (PeproTech, 100-21) for 24h in serum-free medium. [60]

### Cell transfection

All RNA transfections were performed at a final concentration of 50 nM using Lipofectamine 2000 (Invitrogen) according to the manufacturer's instruction. Transfection of plasmid DNA was performed using Lipofectamine LTX reagent (Invitrogen) for SW480 and HCT116 cells and Lipofectamine 2000 for other cells.

### RNA extraction and qRT-PCR assays

Total RNA was extracted from cells or tumors with TRIzol reagent (Invitrogen) according to the manufacturer's instruction. qRT-PCR assays were performed as previously described. [14] U6 and GAPDH were employed as endogenous controls for miRNA and mRNA respectively. The comparative Ct Method (ΔΔCT Method) was used to determine the expression levels of genes. The stem-loop primer for miRNAs was designed according to the published method of Moltzahn *et al*. [61] The primers used for qRT-PCR are shown in Supplementary Table S2.

### Western blot analysis

Total protein was extracted using TRIzol reagent (Invitrogen) as recommended. Protein precipitation was lysed in the buffer containing 1% DTT, 4% CHAPS, 7 M urea, 2 M thiourea and 2% ampholine. Equal total protein extracts were loaded and separated by SDS-polyacrylamide gel electrophoresis (SDS-PAGE), transferred onto Protran™ nitrocellulose membranes (Whatman). The following antibodies were used for western blot: β-catenin (CST, 9582S), p-β-catenin (CST, 9561S), LEF1 (CST, 2230S), TCF4 (CST, 2569S), EP300 (SCB, sc-584), CREB1 (CST, 9197S), E-cadherin (CST, 3195S), ZO-1 (Invitrogen, 40-2200), vimentin (CST, 5741S), α-tubulin (CST, 2144S) or GAPDH (CST, 2118S). GAPDH or α-tubulin was used as the loading control.

### Immunofluorescence analysis

Cells cultured on coverslips were fixed with 4% Paraformaldehyde and incubated with antibodies against E-cadherin (CST, 3195S, 1:50), ZO-1 (Invitrogen, 40-2200, 1:50) or vimentin (CST, 5741S, 1:25), then stained with secondary antibodies (Alexa Fluor® 488, donkey anti-rabbit, Invitrogen, A-21206, 1:400) and nuclear counter-staining with Hoechst 33342 (Life technologies, H1399). Fluorescent images were examined using Zeiss 7 DUO NLO (Leica Microsystems, Bannockburn, IL, USA).

### Immunohistochemistry assay

For Immunohistochemistry (IHC), tissues were fixed in 4% formalin for 1 day and followed by paraffin embedding. 4 μm thick serial sections were used for HE staining or IHC-IF. For IHC-IF, primary antibody was used against GFP (Abcam, ab13970) and Goat Anti-Chicken IgY H&L (Alexa Fluor® 488, ab150173) was used as secondary antibody. Then the fluorescence was detected with Zeiss AxioObserver.Z1 (Carl Zeiss).

### Animal studies

All procedures for the experiments involving mice were performed in accordance with the Guidelines for the Care and Use of Laboratory Animals (NIH publications Nos. 80-23, revised 1996), and according to the ethical principles for experiments on animals. HCT116-miR-150 or HCT116-NC stable cells (1.5×10^6^) were suspended in 200μl 1×PBS and then injected subcutaneously into the dorsal flank of 5-week-old athymic nude mice (BALB/c-nu, n=10/group, Guangdong Medical Laboratory Animal Center, China). The mice were sacrificed 35 days after injection. The primary tumors and livers of mice were dissected, fixed in 4% formalin, and then embedded in paraffin.

### Statistical analysis

Quantitative data were presented as the mean ± the standard error of the mean (SEM) from a minimum of three independent experiments. Comparisons between two groups were analyzed using the Student's *t*-test with n=3, unless otherwise indicated. Statistical analyses were performed with GraphPad Prism 6 (GraphPad Software Inc., San Diego, CA, USA). p<0.05 was considered to be statistically significant.

## SUPPLEMENTARY MATERIALS AND METHODS














