# Supplementary material for: Wnt/β-catenin pathway transactivates microRNA-150 that promotes EMT of colorectal cancer cells by suppressing CREB signaling
Source: Oncotarget. 2016 Jun 7;7(27):42513–26. doi: 10.18632/oncotarget.9893 (PMC5173152; doi:10.18632/oncotarget.9893)
Supplement: Supplementary file 1 [file oncotarget-07-42513-s001.pdf]

Wnt/β-catenin pathway transactivates microRNA-150 that promotes EMT of colorectal cancer cells by suppressing CREB signaling

SUPPLEMENTARY DATA

MATERIALS AND METHODS

RNA oligoribonucleotides

miRNA mimics and inhibitors for miR-150-5p and negative control were obtained from Ambion (Austin, TX, USA). miRNA mimics are small, chemically modified RNAs that mimic the function of endogenous miRNAs. miRNA inhibitors are small, chemically modified single-stranded RNA molecules designed to inhibit miRNA function. The short interfering RNA for human β-catenin (M-003482-00-0005), LEF1 (M-015396-00-0005), EP300 (M-003486-04-0005), CREB1 (M-003619-01-0005) and the negative control (D-001206-14-05) were purchased from Dharmacon (Lafayette, CO, USA).

Vectors construction

The plasmid pCAGGS-HA-LEF1 was a gift from Mitsuyasu Kato (Institute of Basic Medical Sciences, University of Tsukuba, Tsukuba, Japan). The pcDNA3 vector (Invitrogen) was also used for generation of β-catenin, dnTCF4 and CREB1 expression vectors. And in order to construct the constitutively activated β-catenin, double mutations of Ser33 and Ser37 to tyrosine and alanine respectively had been carried out with Multipoints Mutagenesis Kit (TaKaRa, Shiga, Japan). The genomic fragment encoding miR-150 precursor (~ 360 bp) was cloned into pcDNA6.2 (K4936-00, Invitrogen) and pLSNCG for transient and stable transfection, respectively. [1] Various DNA fragments upstream of pre-miR-150 were amplified from SW480 genome and inserted into pGL4.11 (Promega, Madison, WI, USA) in the sense orientation relative to the luciferase coding sequence. And multipoints Mutagenesis Kit (TaKaRa) was used to generate the TBE mutation vector. To identify targets, we synthesized 58-nt DNA oligos containing the putative binding site for miR-150, and the mutated sequence from the 3'UTR of EP300 and CREB1, and then these oligos were annealed and cloned into the psiCHECK-2 vector (Promega). Cignal 45-pathway reporter arrays, Wnt pathway reporter and CREB pathway reporter that measure the activity of the corresponding pathway were purchased from Qiagen (Qiagen, Hilden, Germany). In order to kncokout of CREB1 in HCT116 cells, two gRNAs were designed based on CRISPR gRNA Design tool (DNA2.0). For each single gRNA cloning, we synthesized the sense and antisense oligos from Sangon (Shanghai, China) and annealed the two oligos and then ligated into PX462 (a gift from Jianyou Liao, Research Center of Medicine, Sun Yat-sen Memorial Hospital, Sun Yat-sen University, Guangzhou, China, Addgene, #62987), named PX462-C1 and PX462-C2, respectively. The sgRNA transcription unit (containing U6 promoter and the sgRNA) of PX462-C2 was PCR-amplified by Phanta Max Super-Fidelity DNA Polymerase (Vazyme, China) and subcloned into the *Xba* I and *Kpn* I sites of PX462-C1 vector to generate a dual gRNA vector PX462-C1C2. The primers used for vector construction are listed in Supplementary Table S1.

Luciferase reporter assays

Both forward and reverse transfection methods have been applied for luciferase reporter assay. And 45-pathway assay is performed with reverse transfection, forward transfection is employed for promoter assay, pathway assay and miRNA targets validation assay. For forward transfection, cells are plated in 48-well plate 24 hours before transfection. 48 hours post-transfection, cell extracts were assayed for luciferase activity using the Dual-Luciferase Reporter Assay Systems (Promega) according to the manufacturer's instruction. Reverse transfection involves plating cells into the transfection complexes in a one day procedure. For promoter assay and 45-pathway assay, the Renilla luciferase was used as a control to normalize luminescence levels. While, for miRNA targets validation assay, results were presented after normalization to Firefly luciferase activity.

Migration and invasion assays

For HCT116 and RKO migration and invasion assay, the cells were suspended in 200 ul medium without FBS after 24 h post-transfection of mimics or siRNAs, and then were seeded into upper chamber of transwell inserts (8uM pore size, costar) with or without coated Matrigel (R&D System, Minneapolis, MN). The lower chamber of the transwell was filled with 750ul medium supplemented with 10% FBS which function as a chemoattractant. After 24h incubation in 37°C, cells that migrated or invaded to the lower surface of the insert membrane were fixed in methanol and stained by 0.1% crystal violet. For SW480

migration and invasion assay, the cells were treated with 20 ng/ml TGFβ in serum-free medium for 24h after 16h post-transfection of inhibitor. Then the cells were placed into the upper chamber with the non-coated or Matrigel-coated membrane, which was diluted with 200 ul serum-free culture medium. After the cells were incubated for 48 h at 37°C in a humidified incubator, the cells adhering to the lower surface were fixed in methanol and stained by 0.1% crystal violet. The number of cells that have migrated or invaded were captured under Zeiss AxioObserver.Z1 (Carl Zeiss) at a magnification of 50×. And cell counting was processed by Image Pro-Plus software.

5' and 3' RACE

5'-Full RACE kit (TaKaRa) was used with some modifications. Firstly, poly A+ RNA was purified from total RNA of SW480 cells using the Oligotex mRNA Kit (QIAGEN) according to the manufacturer's instructions. Secondly, reverse transcription was performed using SuperScript III Reverse Transcriptase (Invitrogen). 3' RACE assay was performed according to the manufacturer's instruction of 3'-Full RACE Kit (TaKaRa). Primers are listed in Supplementary Table S3.

ChIP assay

SW480 cells were cross-linked with 1% formaldehyde (Sigma) in new culture medium for 10 minutes and the reaction quenched with addition of glycine to a final concentration of 0.125 M at room temperature for 5 minutes. Cells were rinsed with cold PBS twice and scraped from the dishes. Pellet cells were resuspended in cell lysis buffer (20mM Tris-HCl at pH 8.0, 85 mM KCl, and 0.5% NP-40) and incubated on ice for 15 minutes, vortexing the cell suspension briefly every 5 minutes to facilitate the release of the nuclei. Nuclear lysis buffer containing protease inhibitor mixture (Complete Mini tablets, Roche Diagnostics) was used to lyse the nuclei at a concentration of 1×10^7 cells/ml. After sonication, the chromatin lysates were divided into 100 µl aliquots. Each 100 µl sonicated chromatin (1×10^6 cells) was diluted in 900 µl ChIP dilution buffer, and pre-cleared with 60 µl Protein G Magnetic bead (Invitrogen). 10 µl (1%) of the supernatant was reserved as Input. Cleared samples were immunoprecipitated over-night in IP buffer with 2 µg of anti-β-catenin (BD, 610154), 2 µg of anti-LEF1 (Millipore, 17-604) or 2 µg negative control antibody IgG. Immune complexes were successively washed one time with Low Salt Wash Buffer, one time with High Salt Wash Buffer, one time with LiCl Wash Buffer, two times with TE Buffer for 5 minutes. The precipitated chromatin was eluted and reversed cross-linked in ChIP Elution Buffer containing proteinase K two hours at 65°C. The DNA was recovered and purified using Qiagen PCR purification kit. The semi-quantitative PCR was performed to analyse the immunoprecipitated DNA. SP5 and MyoD acted as positive and negative control respectively. Primers are listed in Supplementary Table S4.

EMSA

For EMSA, total nuclear extracts of SW480 cells were prepared using Nuclear and Cytoplasmic Protein Extraction Kit (Beyotime Biotechnology, P0027) according to the manufacturer's instructions. Wild-type TBE and mutated TBE oligonucleotides were γ-^{32}P-end-labeled (PERKINELMER) with T4 polynucleotide kinase. Subsequently, EMSA was conducted using an EMSA/Gel-Shift kit (Beyotime Biotechnology, GS002) according to the manufacturer's instructions. For competition analyses, unlabeled competitor probes were added to the reaction in 10- and 50-fold molar excess over the labelled Wild-type TBE and mutated TBE oligonucleotides. For the supershift experiments, 1 µg of anti-LEF1 antibody (Cell signaling technology, #2230) was added. As a negative control, we performed a parallel supershift experiments with normal rabbit IgG (Cell signaling technology, #2729). The DNA-protein complexes were separated in 5% polyacrylamide gels (in 0.5×Tris-buffered EDTA buffer) at 4 °C and 20 V/cm, and the gel was dried and visualized by autoradiography (Typhoon, GE). DNA oligonucleotides are listed in Supplementary Table S5.

Knockout of CREB1

The CRISPR/Cas9 Nickase system was used to construct the HCT116 KO-CREB1 cells. The HCT116 cells were transfected with PX462-C1C2 using Lipofectamine 2000 and cultured in the presence of puromycin (3 µg/mL) for 48h after transfection. Single-cells clones were generated by limiting dilution assays and tested for knockout of CREB1 expression by Western blot.

REFERENCE

1. Xu H, He JH, Xiao ZD, Zhang QQ, Chen YQ, Zhou H, et al. Liver-enriched transcription factors regulate microRNA-122 that targets CUTL1 during liver development. Hepatology 2010, 52: 1431-1442.

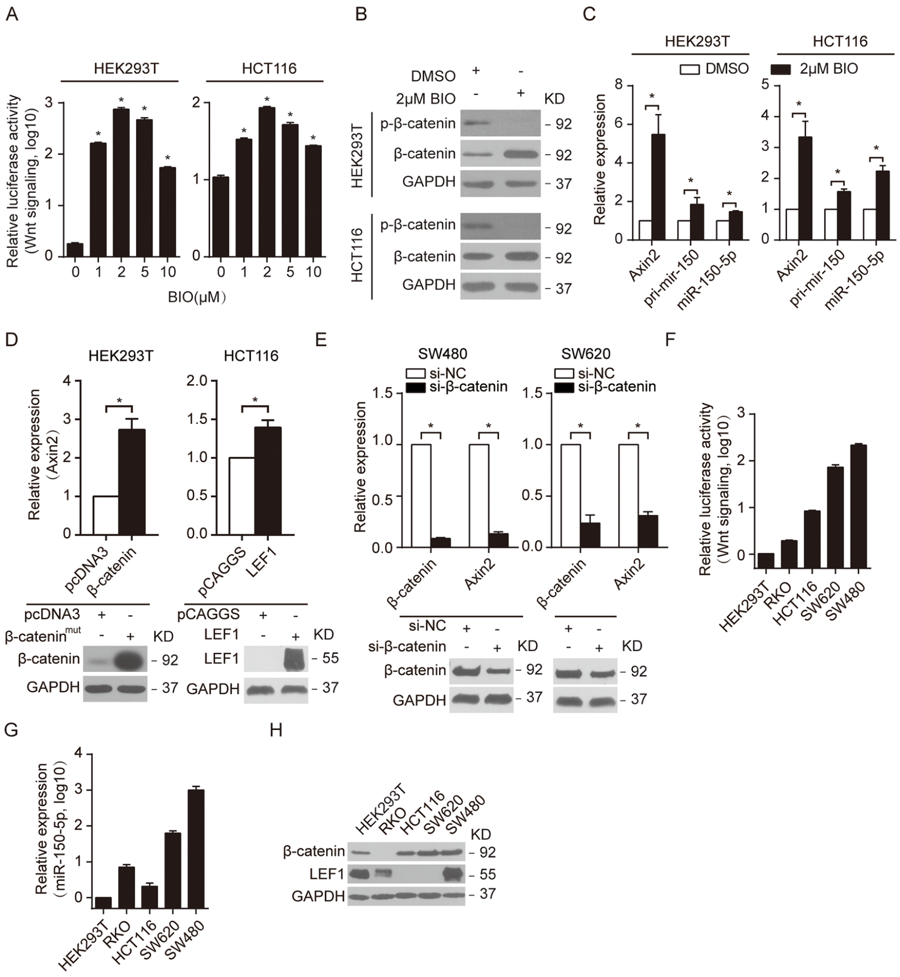

Supplementary Figure S1: Wnt signaling pathway directly regulates miR-150 expression. A-C. Wnt pathway reporter assay **A.**, β-catenin and p-β-catenin Western blot **B.** and Axin2, pri-miR-150 and miR-150-5p qRT-PCR **C.** in HCT116/HEK293T cells treated with BIO as indicated. **D.** Axin2 mRNA levels were measured by qRT-PCR in cells overexpressing β-catenin (HEK293T cells) or LEF1 (HCT116 cells). **E.** Axin2 mRNA levels were detected by qRT-PCR in SW480 and SW620 cells after transfection of β-catenin-specific siRNA or scrambled siRNA. **F.** Relative Wnt signaling activity was quantified using Wnt pathway reporter in the HCT116, RKO, SW620 and SW480 CRC cell lines as well as in HEK293T cells. The values in the bar graph were normalized to the data obtained in HEK293T cells. **G.** The relative expression of miR-150-5p in different cell lines was determined by qRT-PCR. The data were log10-transformed after being normalized to the U6 internal control and the HEK293T cell line control. **H.** The relative expression of β-catenin and LEF1 in different cell lines was determined by Western blot. The qRT-PCR experiments were performed three times with similar results. Error bars represent SEM. *$p < 0.05$ by Student's *t*-test.

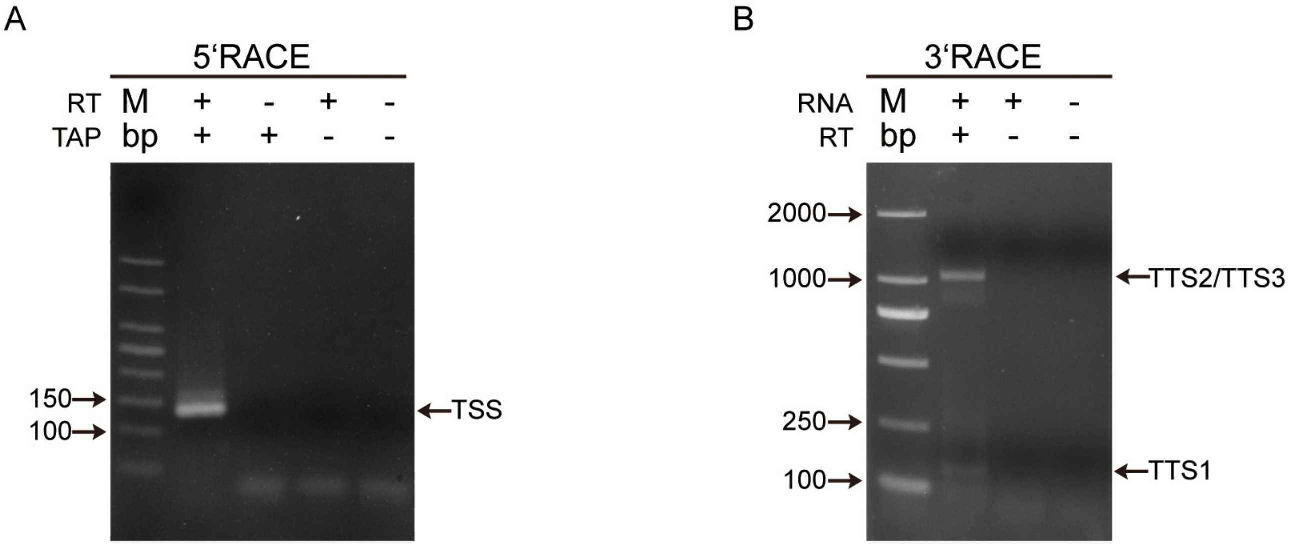

Supplementary Figure S2: Identification of the transcriptional start site (TSS) and transcriptional termination site (TTS) for miR-150 in SW480 cells. A, B. 5'RACE (A) and 3'RACE (B) of miR-150. The TSS and TTSs are marked with arrows. TAP: tobacco acid pyrophosphatase. RT: reverse transcriptase. M: DNA marker with indicated lengths.

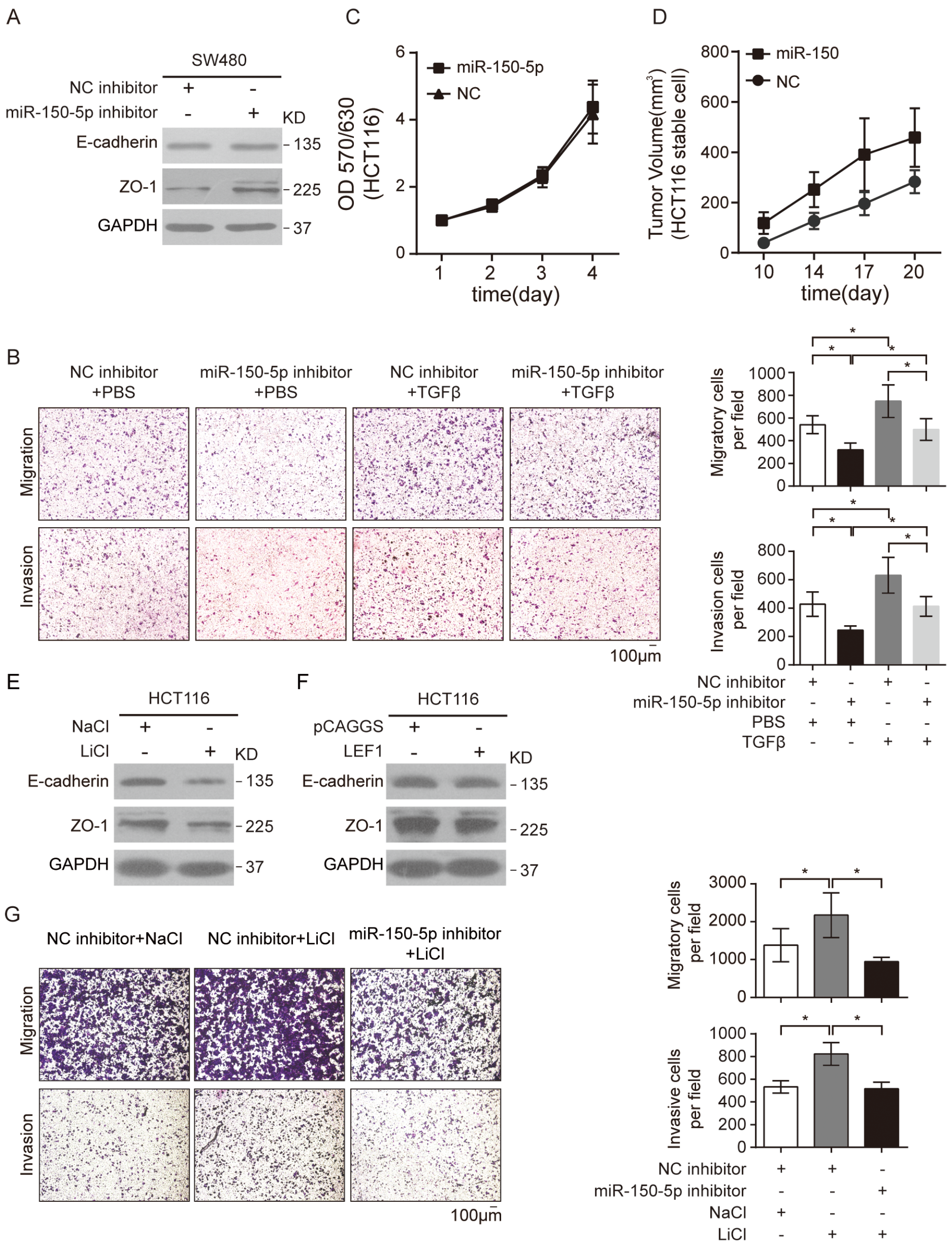

Supplementary Figure S3: MiR-150 is involved in EMT, invasion and migration of SW480 cells. A. Western blot analysis showing the effect of miR-150 inhibition on E-cadherin and ZO-1 expression. GAPDH was used as a loading control. **B.** Inhibiting miR-150-5p blocked TGFβ induced invasion and migration in SW480 cells. The mean number of cells per visual field was determined in three randomly selected visual fields per chamber (right panel). **C.** The effects of miR-150 overexpression on tumor growth kinetics of primary subcutaneous HCT116 tumor xenografts in mice. **D.** MTT showing the effects of miR-150 overexpression on the proliferation of HCT116 cells *in vitro*. **E, F.** Western blot analysis demonstrating that the decreased amount of E-cadherin and ZO-1 expression after LiCl treatment or LEF1 overexpression in HCT116 cells. GAPDH was used as a loading control. **G.** Transwell migration and invasion assays using HCT116 cells after transfection with miR-150-5p inhibitors/NC and treat with LiCl/NaCl. Representative images are shown in the left panel. The mean number of cells per visual field was determined in three randomly selected visual fields per chamber (right panel). MTT and transwell assays were performed in triplication. Error bars represent SEM. *$p < 0.05$ by Student's *t*-test.

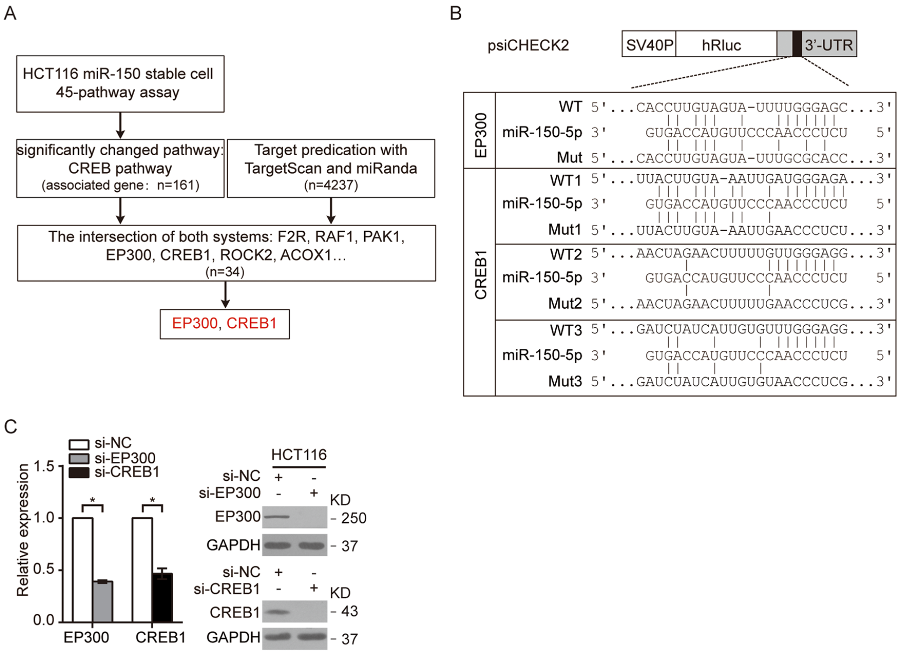

Supplementary Figure S4: miR-150 repressed the CREB pathway by directly targeting EP300 and CREB1. A. Schematic for the identification of miR-150 targets. The putative targets were obtained by identifying the overlap between the CREB signaling pathway-associated genes (KEGG Orthology) and the software-predicted targets. **B.** Schematic of the wild-type (WT) and mutant (Mut) EP300 and CREB1 3'UTR reporter constructs. The alignment between miR-150-5p and the predicted 3'UTR binding sites were shown. **C.** qRT-PCR (left) and Western blot (right) analyses of the mRNA and protein expression of EP300 and CREB1 in HCT116 cells after transfection with siRNA against EP300 or CREB1 or with negative control (NC). The qRT-PCR experiments were performed three times with similar results. Error bars represent SEM. *$p < 0.05$ by Student's t-test.

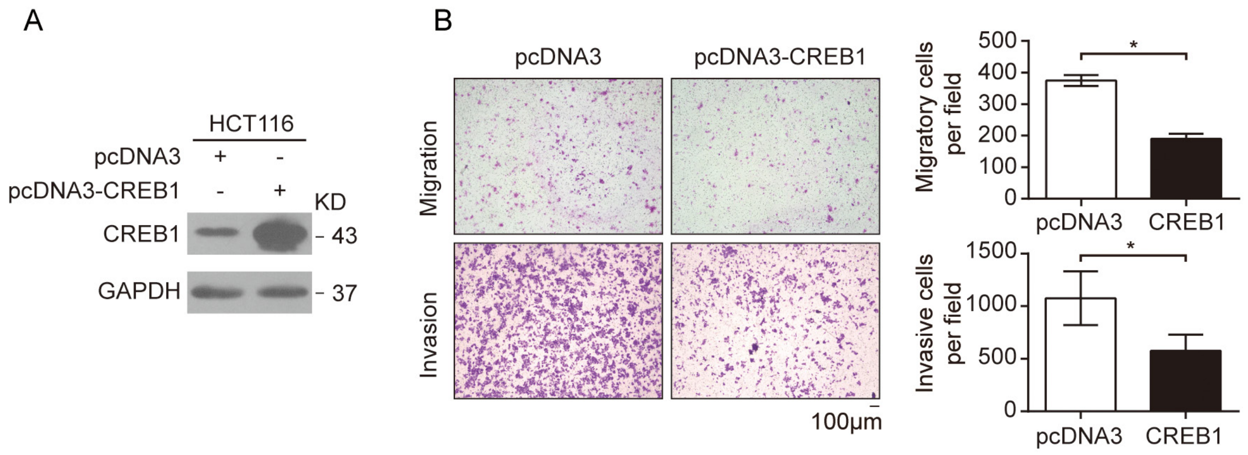

Supplementary Figure S5: Overexpression of CREB1 inhibited the invasion and migration of HCT116 cells. A. Western blot analysis showing the overexpression effect of CREB1 in HCT116 cells. GAPDH served as a loading control. **B.** Migration and invasion assays in HCT116 cells after CREB1 overexpression. Representative images are shown in the left panel. The mean number of cells per visual field was determined in three randomly selected visual fields per chamber (right panel). The transwell results presented are an average of at least three independent experiments. Error bars represent SEM. *$p < 0.05$ by Student's *t*-test.

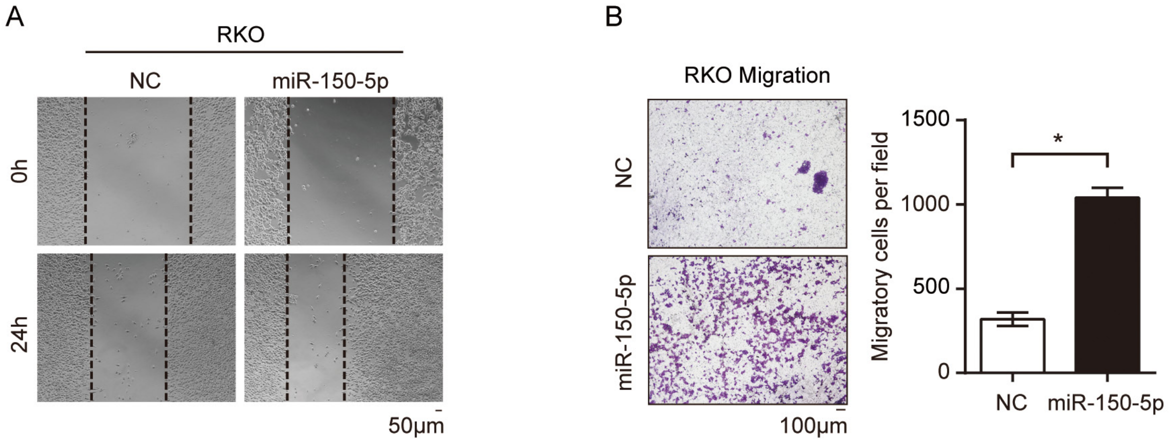

Supplementary Figure S6: Overexpression of miR-150 promotes the migration of RKO cells. A, B. Effects of miR-150-5p mimics on the migration of RKO cells as evidenced by wound-healing assays (A) and transwell assays (B).

Supplementary Table S1: Primers used for vectors construction

See Supplementary File 1

Supplementary Table S2: Primers used for reverse transcription and real-time PCR

See Supplementary File 2

Supplementary Table S3: Primers used for 5'RACE and 3'RACE

See Supplementary File 3

Supplementary Table S4: Primers used for ChIP

See Supplementary File 4

Supplementary Table S5: DNA oligonucleotides used for EMSA

See Supplementary File 5

Supplementary Table S6: miRNA list for Human and mouse conserved miRNA qPCR array

See Supplementary File 6
